# Superinfection by Discordant Subtypes of HIV-1 Does Not Enhance the Neutralizing Antibody Response against Autologous Virus

**DOI:** 10.1371/journal.pone.0038989

**Published:** 2012-06-14

**Authors:** Luzia M. Mayr, Rebecca L. Powell, Johnson N. Ngai, William A. Takang, Arthur Nádas, Phillipe N. Nyambi

**Affiliations:** 1 Department of Pathology, New York University School of Medicine, New York, New York, United States of America; 2 Department of Microbiology, New York University School of Medicine, New York, New York, United States of America; 3 Serology Unit, Medical Diagnostic Center, Yaounde, Cameroon; 4 Department of Obstetrics and Gynaecology, University of Yaounde Teaching Hospital, Yaounde, Cameroon; 5 Institute of Environmental Medicine, New York University School of Medicine, New York, New York, United States of America; 6 Veterans Affairs New York Harbor Healthcare Systems, New York, New York, United States of America; Karolinska Institutet, Sweden

## Abstract

Recent studies have demonstrated that both the potency and breadth of the humoral anti-HIV-1 immune response in generating neutralizing antibodies (nAbs) against heterologous viruses are significantly enhanced after superinfection by discordant HIV-1 subtypes, suggesting that repeated exposure of the immune system to highly diverse HIV-1 antigens can significantly improve anti-HIV-1 immunity. Thus, we investigated whether sequential plasma from these subjects superinfected with discordant HIV-1 subtypes, who exhibit broad nAbs against heterologous viruses, also neutralize their discordant early autologous viruses with increasing potency. Comparing the neutralization capacities of sequential plasma obtained before and after superinfection of 4 subjects to those of matched plasma obtained from 4 singly infected control subjects, no difference in the increase in neutralization capacity was observed between the two groups (p = 0.328). Overall, a higher increase in neutralization over time was detected in the singly infected patients (mean change in IC_50_ titer from first to last plasma sample: 183.4) compared to the superinfected study subjects (mean change in IC_50_ titer from first to last plasma sample: 66.5). Analysis of the Breadth-Potency Scores confirmed that there was no significant difference in the increase in superinfected and singly infected study subjects (p = 0.234). These studies suggest that while superinfection by discordant subtypes induces antibodies with enhanced neutralizing breadth and potency against heterologous viruses, the potency to neutralize their autologous viruses is not better than those seen in singly infected patients.

## Introduction

The importance of neutralizing antibodies (nAbs) was initially shown in various non-human primate models, where passive immunization with nAbs was able to protect against SIV and SHIV-1 infection [1]–[8]. Whilst the ability of nAbs to protect against HIV-1 infection in humans still remains to be defined in more detail, the emergence of nAbs during the course of HIV-1 infection is a critical element of the host humoral immune response against the virus [9]–[13]. Within a few months of HIV-1 infection, nAbs against the autologous virus develop [9]–[11], [14]–[18]. Anti-HIV-1 nAbs in the infected host are polyclonal and they target different epitopes on the viral envelope glycoproteins gp120 and gp41 [19]–[28]. Over the course of acute to chronic infection the immune response matures, leading to the development of more potent antibodies that neutralize early autologous virus. In response to the immune pressure nAbs exert on the virus, escape mutants appear. As a consequence, the potency of nAbs against early autologous viruses increases over time until the response gradually wanes as the virus evolves and recognition of early virus fades. These well-documented successive waves result in nAbs from a specific time point being able to neutralize autologous virus from months earlier, but not concurrent circulating variants [10]–[13], [29]–[31]. Whether or not there are distinct patterns of neutralization of autologous viruses by antibodies from individuals infected with one (singly infected) or two or more (superinfected) HIV-1 subtypes remains to be studied. Furthermore, not much is known on the evolution of nAb responses to early autologous viruses in patients superinfected with discordant or concordant HIV-1 subtype strains [32]–[33].

Superinfection, the concomitant or sequential infection with two or more genetically distinct HIV-1 strains, was shown to occur frequently in communities where diverse HIV-1 subtypes co-circulate and, through reverse transcriptase template switching between two viral RNAs, can result in the generation of recombinant virus strains [34]–[37]. Superinfection provides a unique opportunity to examine how the host immune response is affected when challenged by diverse HIV-1 antigens, particularly with regards to any impact on the induction of nAbs, and can also serve as a natural model for vaccine trial through immunization by concordant or discordant immungens. Previously, our lab and others reported that superinfection by genetically discordant HIV-1 subtypes generated broad and potent nAb activity against heterologous viruses, increasing both potency and breadth of the anti-HIV-1 nAb-response [38]–[39]. This suggested that superinfection strengthens the immune response against heterologous viruses and that vaccines incorporating divergent immunogens may induce more broad and potent nAbs than monovalent ones. However, it remains unknown if superinfection with discordant HIV-1 subtypes also induces antibodies in their host that potently neutralize their early autologous viruses in addition to heterologous nAbs. Therefore, in order to determine whether antibodies in individuals superinfected with discordant viruses that potently and broadly neutralize heterologous viruses also exert potent neutralizing capacities to their infecting early autologous viruses, we tested the nAb activities of sequential plasma samples against autologous pseudotyped viruses generated from the initial and superinfecting virus variants.

## Results

### Neutralization Patterns of the Early Autologous Viruses in Individuals Superinfected with Discordant HIV-1 Subtypes

Two sequential plasma samples from subjects who were identified to be superinfected with two discordant HIV-1 subtypes were tested in neutralization assays to determine the pattern and potency of neutralization of their early autologous viruses. A total of 4 subjects including CMNYU107, CMNYU6518, CMNYU129, and CMNYU6544 were studied. HIV-1 pseudoviruses of the respective initial and superinfecting virus strains were generated via PCR of *gp120* (HXB2 location 6219–7787) using plasma that was collected before superinfection and plasma from the time point of detection of superinfection (Table 1). Each of the pseudotyped viruses were tested in neutralization assays against two-fold serial dilutions (1∶20–1∶2560) of autologous patient plasma obtained before (first plasma) and after (last plasma) superinfection (Table 2).

**Table 1:** Data of HIV-1 viruses infecting study subjects.

Subject ID		Isolated at study month	Subtype
*Superinfected*			
**CMNYU107**	Initial virus	6	CRF36_cpx
	Successive virus	21	G
**CMNYU6518**	Initial virus	0	CRF36_cpx
	Successive virus	15	01_AE- CRF36_cpx URF
**CMNYU129**	Initial virus	0	CRF02_AG
	Successive virus	9	F2
**CMNYU6544**	Initial virus	3	CRF02_AG-F2 URF
	Successive virus	12	CRF02_AG
*Singly-infected*			
**CMNYU133**	Initial virus	0	F2
	Successive virus	15	F2
**CMNYU179**	Initial virus	0	CRF02_AG
	Successive virus	15	CRF02_AG
**CMNYU153**	Initial virus	0	H
	Successive virus	30	H
**CMNYU6542**	Initial virus	3	CRF02_AG
	Successive virus	12	CRF02_AG

#### Neutralization of autologous virus from patient CMNYU107

**Figure 1:**
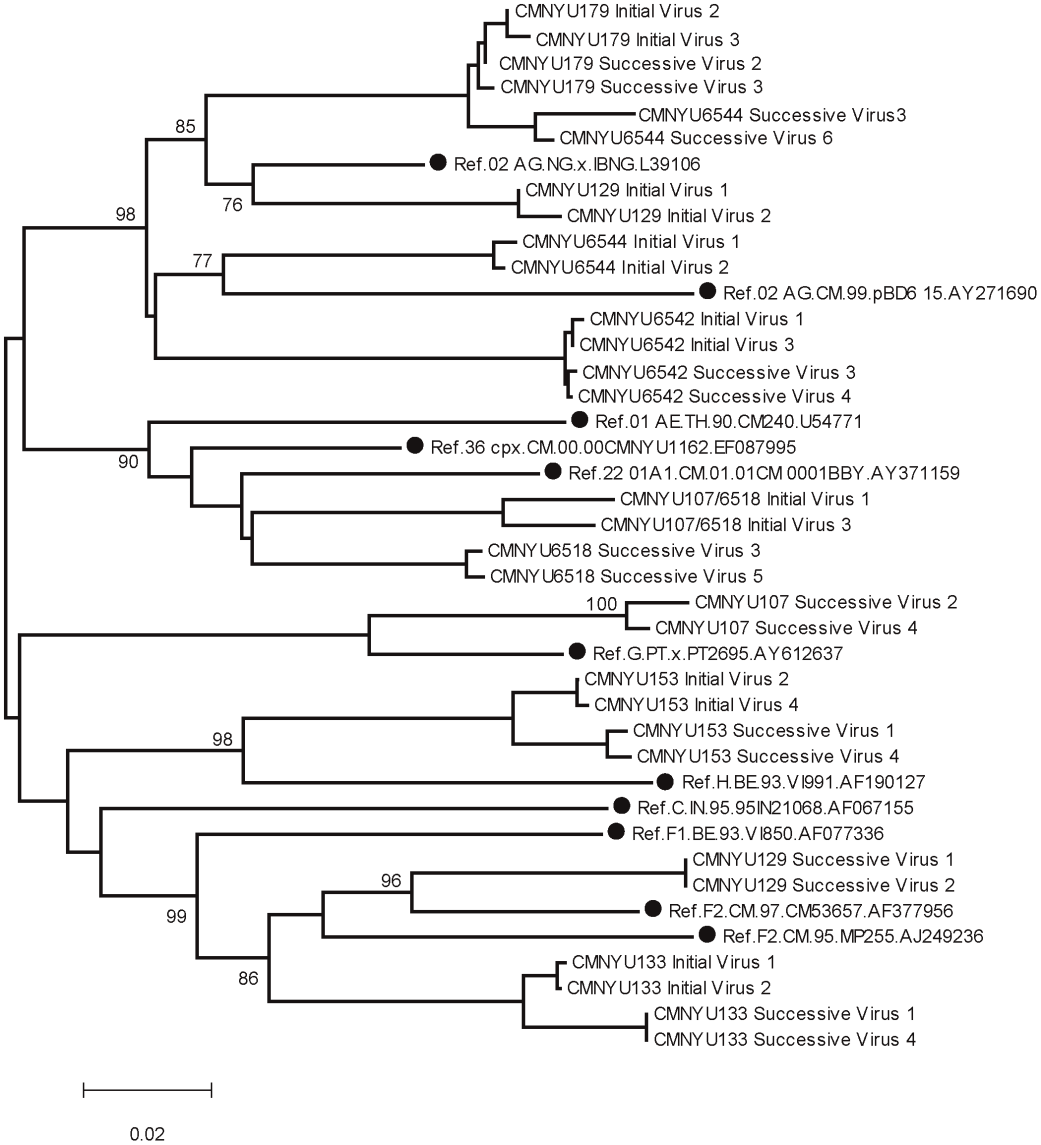
Phylogenetic analysis of *gp120* sequences (HXB2 location 6219–7787) amplified from patient plasma samples and used to construct pseudotyped viruses. Most reference and study subject sequences have been omitted for clarity. Reference sequences are highlighted by black circles.

*gp120* sequences of initial virus variants were amplified from plasma 15 months before superinfection was detected (Table 1). The six initial variants were genetically characterized as CRF36_cpx whilst the six superinfecting variants were defined as subtype G (Figure 1).

**Table 2:** Data of plasma samples from patients used for neutralization.

Subject ID		Plasma from study month	Duration of study (months)	CD4 count	ΔCD4
*Superinfected*					
**CMNYU107**	First plasma	9	27	269	−85
	Last plasma	36		184	
**CMNYU6518**	First plasma	3	57	370	19
	Last plasma	60		389	
**CMNYU129**	First plasma	0	33	603	−266
	Last plasma	33		337	
**CMNYU6544**	First plasma	3	50	266	23
	Last plasma	53		289	
*Singly-infected*					
**CMNYU133**	First plasma	0	21	923	−331
	Last plasma	21		592	
**CMNYU179**	First plasma	0	77	515	−111
	Last plasma	77		404	
**CMNYU153**	First plasma	6	24	290	−11
	Last plasma	30		279	
**CMNYU6542**	First plasma	3	50	709	−110
	Last plasma	53		599	

**Figure 2:**
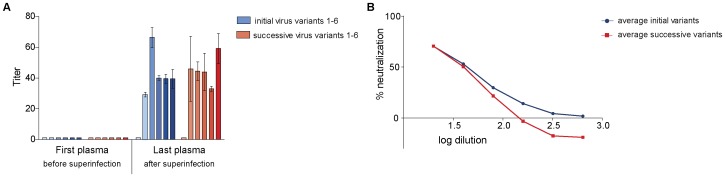
Neutralization of autologous virus variants by serially-diluted plasma obtained from study subject CMNYU107. A: Mean IC_50_ values with standard deviation of initial and superinfecting virus variants. B: Titration of last plasma against initial (mean of initial variants in red) and successive virus variants (mean of successive variants in blue). Only variants that reached 50% neutralization are included.

In our neutralization studies, the plasma sample obtained before superinfection (referred to as first plasma) did not achieve 50% neutralization of the initial or the superinfecting autologous virus variants (Figure 2A). However, the plasma sample obtained 15 months after superinfection (referred to as last plasma) was able to achieve 50% neutralization of 5 of the 6 initial variants (variants 2–6) (mean IC_50_ titer 38) as well as 5 of the 6 superinfecting virus variants with IC_50_ titers ranging from 33 to 59 (mean IC_50_ titer 39) (Figures 2A and 2B). This increase in neutralization by the plasma sample obtained 15 months after superinfection was statistically significant for both initial and superinfecting variants (p = 0.0043).

#### Neutralization of autologous virus from patient CMNYU6518

**Figure 3:**
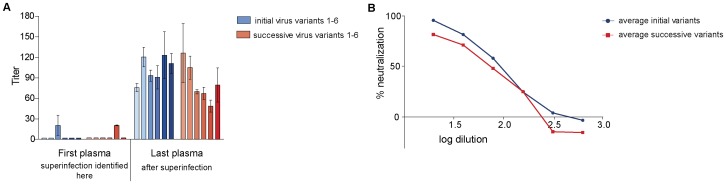
Neutralization of autologous virus variants by serially-diluted plasma obtained from study subject CMNYU6518. A: Mean IC_50_ values with standard deviation of initial and superinfecting virus variants. B: Titration of last plasma against initial (mean of initial variants in red) and successive virus variants (mean of successive variants in blue). Only variants that reached 50% neutralization are included.

Because plasma obtained from this patient prior to superinfection was exhausted in other studies and because those studies revealed that variants were epidemiologically related to the initially infecting variants (subtype CRF36_cpx) from patient CMNYU107, the same pseudovirus variants were also tested in neutralization with subsequent plasma from patient CMNYU6518. The superinfecting variants (n = 6) from this patient (CMNYU6518) were defined as a URF of CRF36_cpx and CRF01_AE [37] (Figure 1). The neutralization potency of two sequential plasma samples obtained from the time point of detection of superinfection (first plasma) and 57 months after detection of superinfection (last plasma) were determined with the pseudotyped viruses from this patient (Table 2). Plasma acquired at the time point of superinfection neutralized only 1 of 6 initial virus variants, exhibiting an IC_50_ titer of 21, and 1 of 6 superinfecting variants with an IC_50_ titer of 19.5 (Figure 3A). The later plasma sample (obtained 57 months after superinfection) was able to neutralize all 6 initially-infecting variants, exhibiting significantly increased IC_50_ titers of 76–123 (mean IC_50_: 102, p = 0.0005). Fifty percent neutralization was also reached against all 6 superinfecting variants using the last plasma sample, a significant increase from the plasma obtained at time of detection of superinfection (mean IC_50_: 82, p = 0.0005) (Figures 3A and 3B).

#### Neutralization of autologous virus from patient CMNYU129

**Figure 4:**
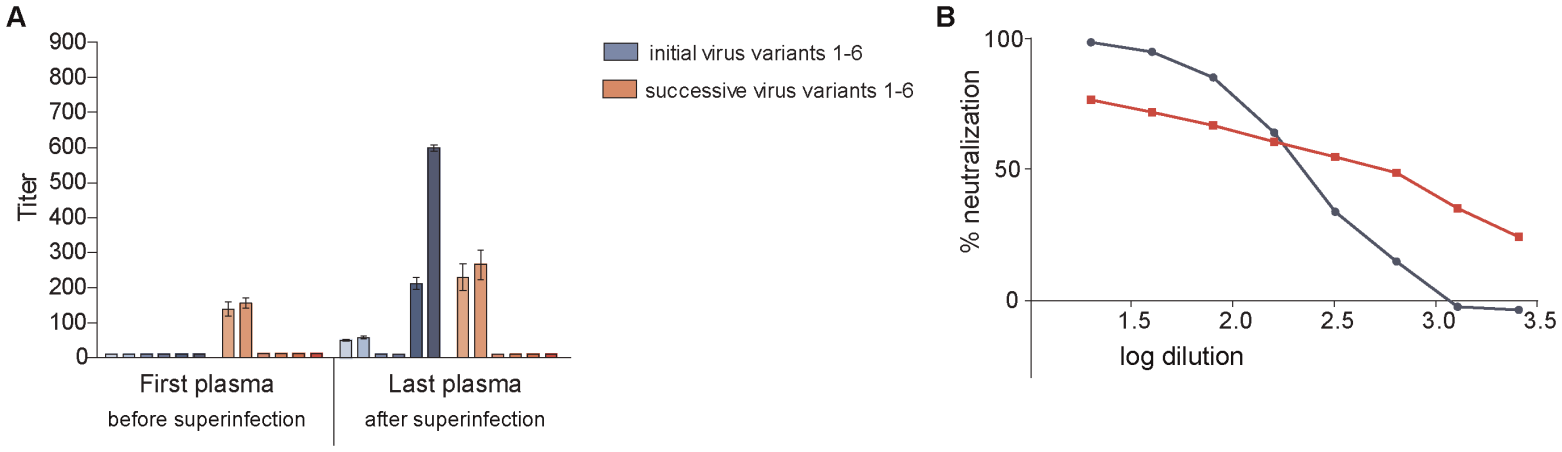
Neutralization of autologous virus variants by serially-diluted plasma obtained from study subject CMNYU129. A: Mean IC_50_ values with standard deviation of initial and superinfecting virus variants. B: Titration of last plasma against initial (mean of initial variants in red) and successive virus variants (mean of successive variants in blue). Only variants that reached 50% neutralization are included.

g*p120* sequences from 6 initially infecting virus variants were amplified from plasma obtained 9 months before superinfection was detected (Table 1) and phylogenetically characterized as CRF02_AG while all the superinfecting variants were characterized as sub-subtype F2 (Figure 1). Plasma obtained 9 months before superinfection was unable to neutralize the initial variants. However, this plasma sample achieved 50% neutralization against two variants of the superinfecting virus with IC_50_ titers of 136 against variant 1 and 152 against variant 2 (Figure 4A). The last plasma sample, obtained 24 months after superinfection, neutralized 4 of 6 initial variants (IC_50_ titers of 49, 58, 212, and 599) and 2 of 6 superinfecting variants (IC_50_ titers of 230 and 266) (Figures 4A and 4B). The increase in neutralization of the initial virus variants by the last plasma sample was significant (p = 0.0183) whilst that of the successive virus variants was not significant (p = 0.839).

#### Neutralization of autologous virus from patient CMNYU6544

**Figure 5:**
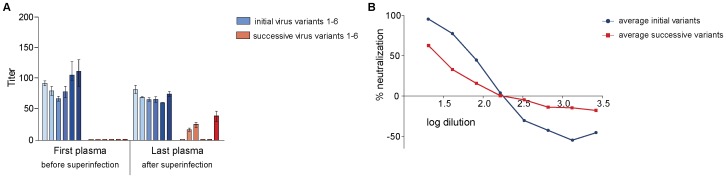
Neutralization of autologous virus variants by serially-diluted plasma obtained from study subject CMNYU6544. A: Mean IC_50_ values with standard deviation of initial and superinfecting virus variants. B: Titration of last plasma against initial (mean of initial variants in red) and successive virus variants (mean of successive variants in blue). Only variants that reached 50% neutralization are included.

Initial variants (n = 6) amplified from patient plasma nine months before detection of superinfection (Table 1) were genetically characterized as a URF of CRF02_AG/F2 while the superinfecting variants were characterized as CRF02_AG with ∼15% distance from the initial variants (Figure 1). The neutralization studies revealed that the first plasma (collected nine months before detection of superinfection) was able to achieve 50% neutralization of all 6 initial variants (mean IC_50_ titer: 98), whilst none of the superinfecting virus variants were neutralized (Figure 5A). The last plasma sample (obtained 41 months after detection of superinfection) exhibited IC_50_ titers of 65–88 against the 6 initial variants (mean IC_50_ titer: 75). This last plasma also achieved 50% neutralization of 3 of the 6 superinfecting variants (variant 2, 3 and 6) at IC_50_ titers of 19, 28 and 43, respectively (Figures 5A and 5B).

### Neutralization Patterns of the Early Autologous Viruses in Individuals with Single HIV-1 Subtype Infection

Two sequential plasma samples from patients from the same cohort chronically infected with HIV-1 who were identified to be singly infected with one subtype virus were also tested in neutralization assays to determine the pattern and potency of neutralization of their early autologous viruses. A total of 4 subjects including CMNYU133, CMNYU179, CMNYU153 and CMNYU6542 were studied; *gp120*s of the infecting HIV-1 variants were amplified (HXB2 location 6219–7787) from their plasma samples at two different time points, matching the timing of the superinfected study subjects (Table 1). The amplified *gp120*s were used to produce pseudotyped viruses and tested in neutralization assays with two-fold serial dilutions (1∶20–1∶2560) of autologous patient plasma (Table 2).

#### Neutralization of autologous virus from patient CMNYU133

**Figure 6:**
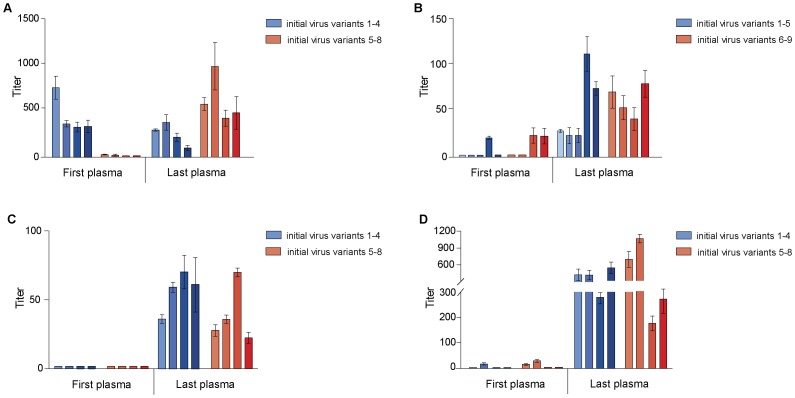
Neutralization of autologous virus variants by serially-diluted plasma obtained from singly infected patients. Mean IC_50_ values with standard deviation of A: study subject CMNYU133. B: study subject CMNYU179. C: study subject CMNYU153. D: study subject CMNYU6542.

The virus variants were amplified from an early (variants 1–4) and a late plasma sample (variants 5–8) (Table 1). Phylogenetic analysis of the *gp120* sequences of all the variants of CMNYU133 revealed clustering with subtype F2 (Figure 1). The first plasma achieved 50% neutralization of its contemporaneous virus variants 1–4 with IC_50_ values of 334–777. A non-significant decline in IC_50_ levels against virus variants 1–4 was detected in the last plasma sample, with a mean IC_50_ titer of 252 (p = 0.076) (Figure 6A). Pseudovirus variants 5–8 could not be neutralized by the first plasma sample. However, the last plasma, obtained 21 months later, was able to achieve 50% neutralization of variants 5–8 (mean IC_50_ titer: 633). This was a significant increase in neutralization (p = 0.00673) (Figure 6A).

#### Neutralization of autologous virus from patient CMNYU179

The virus variants were amplified from an early plasma sample (variants 1–5) and a late plasma sample (variants 6–9) (Table 1). Phylogenetically, the *gp120* sequence of all variants clustered with CRF02_AG (Figure 1). The first plasma sample achieved 50% neutralization of variant 4 (Figure 6B). The last plasma (obtained 77 months later) was able to neutralize all early virus variants (variants 1–5) (IC_50_ titers ranging from 23 to 114, significant increase, p = 0.00184) and all virus variants that were isolated from a later time point plasma (variants 6–9) (IC_50_ titers ranging from 41 to 80, significant increase, p = 0.00673) (Figure 6B).

#### Neutralization of autologous virus from patient CMNYU153

HIV-1 viruses pseudotyped with *gp120* of 8 variants infecting study subject CMNYU153 were amplified (early variants 1–4, later variants 5–8) (Table 1). Phylogenetic analysis revealed clustering of all the variants with subtype H (Figure 1). The first plasma was unable to achieve 50% neutralization of the 8 virus variants (Figure 6C). However, the last plasma sample, obtained 24 months after the first plasma, neutralized variants 1–4 (mean IC_50_ titer: 56, p = 0.00673) as well as variants 5–8 (mean IC_50_ titer: 38), a significant increase from the first plasma (p = 0.00673) (Figure 6C).

#### Neutralization of autologous virus from patient CMNYU6542

HIV-1 viruses pseudotyped with *gp120* sequences of 8 variants infecting study subject CMNYU6542 were amplified (early variants 1–4, later variants 5–8) (Table 1). Phylogenetically, all 8 variants clustered with CRF02_AG (Figure 1). The first plasma sample was able to achieve 50% neutralization of variants 2, 5, and 6, exhibiting IC_50_ titers of 13–27. The last plasma sample, obtained 50 months after the first plasma, exhibited a significantly increased neutralization of all the variants tested (variants 1–4: mean IC_50_ titer: 419, p = 0.00673; variants 5–8: mean IC_50_ titer: 548, p = 0.00673) (Figure 6D).

### Comparison of Neutralization Sensitivity of Plasma Derived *gp120* Pseudotyped Viruses Versus PBMC Isolated Viruses from Patients

**Figure 7:**
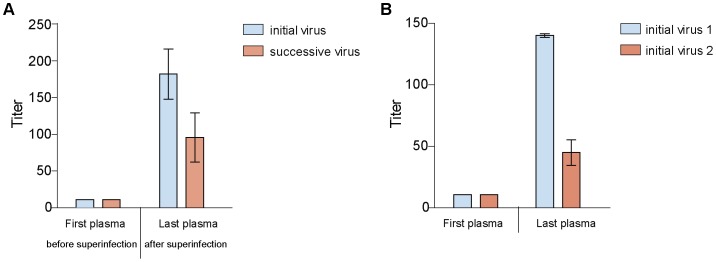
Neutralization of autologous PBMC derived virus by serially-diluted plasma. Mean IC_50_ values with standard deviation of A: study subject CMNYU107 (superinfected). B: study subject CMNYU179 (singly infected).

The pseudoviruses used in our neutralization studies described above bear the *gp120* portion of the envelope derived from the study subjects’ plasma. Therefore the gp120-gp41 envelopes are chimeras that may not display the same epitopes as the native envelope would in its natural form. Anti-gp41 antibodies may be present in the patient plasma that might not recognize the gp41 used for the pseudoviruses. We therefore questioned whether the *gp120* pseudotyped viruses used in our studies would similarly be neutralized like the viruses isolated from patient PBMCs. For this, we isolated the early autologous viruses from two patients (CMNYU107 [superinfected] and CMNYU179 [singly infected]), using the same time points that were used for pseudovirus construction, and compared their neutralization sensitivity with those of the corresponding pseudotyped viruses. In study subject CMNYU107, just as the first plasma sample (obtained 12 months before superinfection) did not neutralize the pseudoviruses, it also did not neutralize the PBMC derived viruses. Furthermore, the last plasma sample acquired 15 months after superinfection) similarly neutralized both the initial and superinfecting pseudovirus variants as well as the PBMC derived initial and superinfecting viruses (Figures 2 and 7A). In patient CMNYU179 the first plasma sample did not neutralize the PBMC derived viruses but neutralized 3 of 9 pseudovirus variants at low level. The last plasma sample of study subject CMNYU179 was able to neutralize all pseudovirus variants and the PBMC derived viruses (Figures 6B and 7B). From this we conclude that the envelopes in the pseudoviruses studied are representative of the *gp160* envelopes in the natural viruses.

### Comparison of IC_50_ Titers of Superinfected and Singly Infected Study Subjects against Autologous Pseudovirus Variants

Neutralization against the initial virus variants increased over time in 3 of the 4 superinfected subjects (range 4-16-fold increase) (Table 3, and Figures 2, 3, 4, 5) and in 3 of the 4 singly infected patients (range 4-35-fold increase) (Table 3, and Figure 6) whilst IC_50_ titers against the successive virus variants increased over time in all the superinfected (range 2-7-fold increase) (Table 3, and Figures 2, 3, 4, 5) and singly infected study subjects (range 4-40-fold increase) (Table 3, and Figure 6). Whilst the increase in neutralization from first to last plasma sample in the superinfected study subjects (mean change in IC_50_ titer from first to last plasma sample: 66.5) was lower than in the singly infected patients (mean change in IC_50_ titer from first to last plasma sample: 183.4), no significant difference in the increase in neutralization capacity against their early autologous viruses was observed between the two groups (p = 0.328).

**Table 3:** Comparison of mean IC_50_ titers of superinfected and singly infected study subjects against autologous pseudovirus variants.

		Initial variants		Successive variants	
Subject ID	Plasma sample	Mean IC_50_	Fold increase	Mean IC_50_	Fold increase
*Superinfected*					
**CMNYU107**	First plasma	10	4	10	4
	Last plasma	38*		39*	
**CMNYU6518**	First plasma	12	9	12	7
	Last plasma	102*		82*	
**CMNYU129**	First plasma	10	16	55	2
	Last plasma	156*		89	
**CMNYU6544**	First plasma	98	0	10	2
	Last plasma	75		20*	
*Singly-infected*					
**CMNYU133**	First plasma	456	0	16	40
	Last plasma	252		633*	
**CMNYU179**	First plasma	12	4	16	4
	Last plasma	53*		61*	
**CMNYU153**	First plasma	10	6	10	4
	Last plasma	56*		38*	
**CMNYU6542**	First plasma	12	35	15	37
	Last plasma	419*		548*	

### Determination of Breadth-Potency Scores

**Figure 8:**
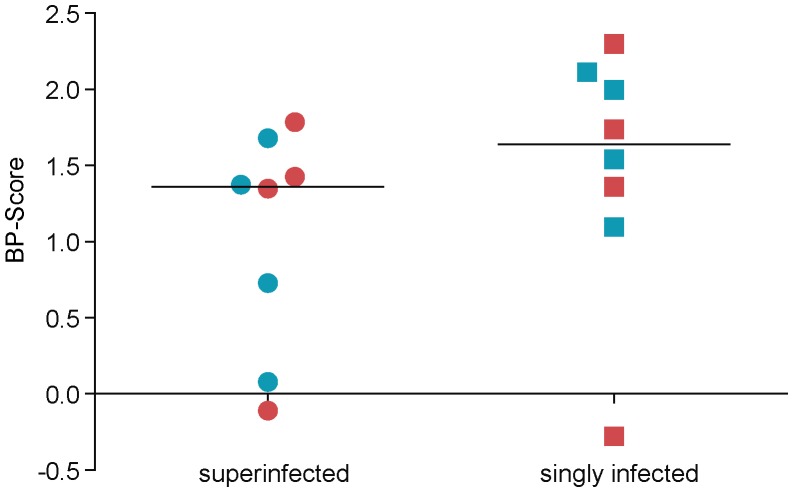
Mean change in B-P Scores in superinfected and singly infected study subjects. Circles and squares represent superinfected and singly infected samples, respectively. Horizontal lines indicate mean values. Red colour indicates the initial virus variants, whilst colour blue indicates the later virus variants.

Breadth-Potency scores (B-P Scores) were calculated in order to be able to compare the neutralization capacity of the singly and superinfected patients’ plasma. The B-P score was computed by averaging all obtained x values, which were calculated by setting x = log10(IC_50_) for the available IC_50_s and by setting x = 0 for viruses that did not neutralize. This is equivalent to multiplying the fraction of viruses neutralized by the mean of the available IC_50_s, which in turn is the area under the B-P curve [40]. First, the neutralization breadth and potency of the respective first and last plasma samples against the initial pseudoviruses were analyzed. These pseudoviruses were called “initial virus variants” in the superinfected study group and “initial virus variants 1–4 or 5″ in the singly infected control subjects. For superinfected and singly-infected study subjects, these initial pseudoviruses were generated from plasma that was isolated an average of 0.25 months before the first plasma sample that was used for neutralizations and an average of 42.6 months before the last plasma sample used for neutralization. There was no significant difference between singly and superinfected study subjects in the time elapsed between plasma samples used for neutralization and the generation of pseudoviruses. The mean change of B-P Scores in the initial virus variants between the last and the first plasma sample tested was 1.112 in the superinfected study subjects (range from −0.110 to 1.784) and 1.280 in the singly infected subjects (range from −0.278 to 2.300). No significant difference in change of B-P Score was observed between singly and superinfected patients (p = 0.886) (Figure 8). Next, the neutralization breadth and potency of the respective first and last plasma samples against the “later” pseudoviruses were analyzed. These pseudoviruses were called “successive virus variants” in the superinfected study group and “initial virus variants 5–8 or 9″ in the singly infected control subjects. These later pseudoviruses were generated from plasma that was isolated an average of 13.125 months after the first plasma sample that was used for neutralizations and an average of 29.25 months before the last plasma sample that was used for neutralization. There was no significant difference between singly and superinfected study subjects in the time elapsed between plasma samples used for neutralization and the generation of pseudoviruses. The mean change of B-P Score between the last and the first plasma sample tested was 0.965 in the superinfected study subjects (range from 0.078 to 1.680) and 1.687 in the singly infected subjects (range from 1.097 to 2.113). No significant difference in change of B-P Score was observed between singly and superinfected patients (p = 0.2) (Figure 8).

### Correlation between the Heterologous and Autologous Neutralization Capacity of Superinfected and Singly Infected Study Subjects

Previously, our lab reported that HIV-1 superinfection by genetically distinct strains correlates with significantly increased potency and breadth of the nAb response against heterologous viruses [38]. The IC_50_ titers of super- and singly infected patients’ plasma against 6 heterologous primary isolates were determined, including Bx08 (subtype B), CMNYU102 (CRF02_AG), CMNYU104 (sub-subtype F2), CMNYU122 (CRF02_AG), CMNYU161 (CRF02_AG), and CMNYU6491 (subtype G) [38]. In 3 of 4 superinfected patients, plasma samples collected after superinfection (referred to as last plasma) exhibited significantly increased IC_50_ titers relative to plasma samples obtained before superinfection (referred to as first plasma). The mean increase in neutralization against heterologous viruses from first to last plasma sample in superinfected patients CMNYU107, CMNYU6518 and CMNYU6544 was 55-fold, 12-fold, and 25-fold, respectively (Table 4) [38]. In comparison, the same samples tested against their respective autologous pseudoviruses on average only achieved a 4-8-fold increase in neutralization (Table 4 and Figures 2, 3, 4, 5). Whilst the singly-infected control subjects exhibited no significant changes in their magnitude of neutralization against heterologous viruses over the study period, a significant increase in mean IC_50_ from first to last plasma sample with all 4 patients’ plasma tested against their respective autologous pseudoviruses was detected (mean increase from first to last plasma sample in IC_50_ titers ranging from 2-37-fold) (Table 4 and Figure 6). These results clearly demonstrate that while superinfection with discordant HIV-1 subtypes enhances the ability to neutralize heterologous viruses, it does not enhance the neutralization of early autologous viruses.

**Table 4:** Comparison of mean IC_50_ titers of superinfected and singly infected study subjects against heterologous and autologous viruses.

Subject ID	Plasma sample	Mean IC_50_ against heterologous viruses (n = 6)	Fold increase	Mean IC_50_ against autologous pseudoviruses (n = 8–12)	Fold increase
*Superinfected*					
**CMNYU107**	First plasma	10	55	10	4
	Last plasma	550*		38*	
**CMNYU6518**	First plasma	38	12	12	8
	Last plasma	459*		92*	
**CMNYU129**	First plasma	10	0	32	4
	Last plasma	10		123	
**CMNYU6544**	First plasma	10	25	54	0
	Last plasma	251*		47	
*Singly-infected*					
**CMNYU133**	First plasma	21	0	236	2
	Last plasma	10		442*	
**CMNYU179**	First plasma	10	0	14	4
	Last plasma	10		57*	
**CMNYU153**	First plasma	13	0	10	5
	Last plasma	15		47*	
**CMNYU6542**	First plasma	16	0	13	37
	Last plasma	12		483*	

IC_50_ titers against heterologous viruses were taken from the following publication: Powell, R. L., T. Kinge, and P. N. Nyambi. 2010. Infection by discordant strains of HIV-1 markedly enhances the neutralizing antibody response against heterologous virus.

### Correlation between Viral Load, CD4 Counts and Neutralization Patterns

While the mean CD4 count of the initial plasma samples obtained from superinfected study subjects (mean CD4 count: 377 cells/µl) was lower than the initial plasma samples from the singly infected control group (mean CD4 count: 609 cells/µl), this difference was not statistically significant (p = 0.2). In the final plasma samples from the singly infected control subjects (mean CD4 count: 469 cells/µl) and the superinfected subjects (mean CD4 count: 300 cells/µl), the mean CD4 counts were also not significantly different (p = 0.2). The mean change in CD4 count for the control group from the first to the last plasma sample was –141 cells/µl, while that of the superinfected group was –77 cells/µl. These changes were not significantly different (p = 0.343) (Table 2). For example, the study duration was similar for superinfected patient CMNYU107 (27 months) and the singly infected patient CMNYU153 (24 months). Both subjects started with comparable CD4 counts (269 and 290 cells/µl, respectively) and experienced a slight drop in CD4 levels over the study period (–85 and –11 cells/µl, respectively). The neutralization pattern of CMNYU107 and CMNYU153 was also comparable, with the initial plasma sample being unable to achieve 50% neutralization of the autologous virus variants and the final plasma sample reaching mean IC_50_ titers of 47 and 38, respectively. Thus, in both subjects the drop in CD4 counts did not impair the neutralizing antibody response. CMNYU133 was the only patient where a decrease in IC_50_ titers against the initial virus variants was observed over time – in this patient the CD4 counts also decreased substantially over the study period (–331 cells/µl). However, in this study subject the nAb response against the later virus variants was increasing over the study period, therefore the drop in CD4 counts cannot be made accountable for the decrease in IC_50_ titers against initial variants. Viral load data for some of the first and all of the last plasma samples has previously been reported and no significant difference was detected between the superinfected and singly infected groups [38].

## Discussion

Previously, we and others reported enhanced nAb responses in individuals superinfected with discordant HIV-1 subtype strains against several HIV-1 heterologous viruses [38]–[39]. Because these previous studies suggested that reinfection with a discordant subtype enhances the patient’s immune response to generate antibodies that neutralize heterologous viruses, we hypothesized that the potency to neutralize early autologous viruses in the superinfected patients would also be increased compared to the singly infected control subjects. Thus, in this current study, sequential plasma samples of the 4 patients superinfected with discordant HIV-1 subtype strains we previously studied were tested for their potency and breadth to neutralize viruses pseudotyped with autologous *gp120*. In comparison, the neutralization capacity of matched plasma from 4 singly infected study subjects against their early autologous pseudoviruses was measured. We observed that the increase in neutralization from first to last plasma sample in the superinfected study subjects (mean change in IC_50_ titer from first to last plasma sample: 66.5) was lower than in the singly infected patients (mean change in IC_50_ titer from first to last plasma sample: 183.4). However, the difference between the two groups was not significant (p = 0.328). Similarly, the calculation of Breadth-Potency Scores confirmed that there was no significant difference in the increase in superinfected and singly infected study subjects (p = 0.234) from first to last plasma sample used for neutralization. Therefore, as illustrated in Table 4, increased potency and breadth to neutralize heterologous viruses by the superinfected patient’s plasma did not translate into increased potency to neutralize early autologous viruses compared to the singly infected study subjects.

It is intriguing to note that while superinfection by discordant HIV-1 subtypes helps to boost the humoral immune response against heterologous viruses [38], it does not boost the response against its early autologous viruses. Previous studies have demonstrated that different HIV-1 subtype strains share conserved antigenic epitopes while also harboring epitopes that are unique to them [41]–[51]. Therefore, the possibility exists that distinct antigenic epitopes from the discordant HIV-1 subtypes infecting these patients broaden the immune response to different viruses. In that case the superinfecting virus may deviate the focus of the immune response from more conserved epitopes as well as reduce the immune pressure on the initially infecting virus strain. This is supported in part by the longitudinal analysis presented here and by others revealing an increased immune response to autologous virus over time in singly infected patients [10]–[11], [29]–[30]. Though not significant, overall the sequential plasma from the singly infected patients tested in this study exhibited a higher neutralization capacity (mean change in IC_50_ titer from first to last plasma sample: 183.4) to their early autologous viruses compared to those from the superinfected patients (mean change in IC_50_ titer from first to last plasma sample: 66.5) (Table 3).

It could be argued that the lack of a better enhancement of the neutralization capacity in the superinfected patients to their early autologous viruses compared to the singly infected patients could be due to a weakened immune system caused by the superinfecting virus. It is important to note that though the patients studied were in their chronic phase of infection, they were all asymptomatic, had been infected with HIV for more than 3 years, and the mean CD4 counts for the superinfected (346 cells/µl) versus the singly infected patients (506 cells/µl) were similar. This suggests that the state of their immune systems were comparable and not weak. Also, if the immune system of the superinfected patients were weaker, the sequential plasma from these same patients would not have exhibited the robust neutralization capacity against heterologous viruses as demonstrated in our previous studies [38]. It is, however, certain that the immune system during acute infection is stronger than during chronic infection. Thus, studies that would examine such responses during acute infection are warranted.

Whether or not superinfection by discordant subtypes would induce a better humoral immune response to its early autologous viruses compared to superinfection by concordant virus subtypes remains to be studied as it would have several implications for vaccine design and strategies. As an example of a potential implication in a therapeutic vaccine strategy, the present results would suggest that challenge of an HIV-1 positive patient with an immunogen genetically divergent from the HIV-1 strain already infecting this patient might lead to deviation of the autologous immune response away from the infecting virus and as a consequence may lead to even faster disease progression. Furthermore, the fact that these superinfected patients do not exhibit an enhanced potency to neutralize their early autologous virus might have implications in disease progression. If the immune response becomes less focused on the initial virus as a result of immune stimulation from a different reinfecting virus, the initial virus might replicate in the host uncontrolled over a period of time. This increase in virus replication of the initial virus would possibly result in further immune stimulation to its epitopes and may then result in deviation of the response to the superinfecting virus - also giving rise to a higher replication of the reinfecting virus. This possible cyclical chain of events might then lead to faster disease progression. However, such studies to examine the impact of superinfection, evolution of viral replication, viral load, nAb patterns, and disease progression need to be carefully examined. While some studies have revealed a rapid disease progression in some patients superinfected with discordant subtypes [16], [52], the number of patients studied are few and studies that examine superinfection and disease progression still remain sparse and incomplete [33], [53]–[55].

The nAb responses against autologous pseudoviruses representing different variants from the patients at each time point were not always uniform, given the genetic variability of the quasispecies (Figures 2, 3, 4, 5, 6). Further study will focus on the sequence analysis of the initial and superinfecting virus variants and factors such as insertions and deletions, point mutations, glycan-shielding, and in some cases the emergence of new recombinant strains within the superinfected patients that might contribute to viral escape from neutralization or a gradual switch of nAb responses to eptiopes on the variants due to antigenic stimulation by a different variant or a recombinant strain [10]–[11], [29]–[31].

Overall, the increased breadth and potency of the nAb response against heterologous viruses by sequential plasma from patients superinfected with discordant HIV-1 subtypes previously reported by us [38] did not correspond with an enhanced potency to neutralize early autologous viruses. While the mechanisms behind this observation remain to be investigated, the results demonstrated here suggest that although antibodies present in superinfected patients may be potent in neutralizing heterologous viruses, their lack of a significantly enhanced neutralizing activity against their autologous viruses does not confer advantages in virus control in the superinfected patients compared to singly infected patients. Because the number of subjects studied may be small, further studies involving larger cohorts of patients superinfected with discordant subtype viruses are warranted to monitor the development of neutralizing antibodies and responses to autologous viruses.

## Materials and Methods

### Ethics Statement

The study was approved by the New York University School of Medicine Institutional Review Board and by the National Ethical Review Board in Cameroon. All volunteers signed written approved informed consent forms prior to participating in the study.

### Study Subject Data

The blood samples analyzed in this study were collected in calendar years 2001–2008 in Yaounde, Cameroon, at 3 to 6 month intervals from a cohort of HIV+, chronically infected individuals. This cohort has been described extensively in our previously published work [37]–[38], [56]. The subjects of interest to the present study were chronically infected with HIV-1 and later determined to be superinfected with discordant HIV-1 subtypes and include: CMNYU107, CMNYU6518, CMNYU129, and CMNYU6544. All cohort study subjects were antiretroviral drug-naïve. The subjects were selected based on their identification as superinfected at an interim study time point and their HIV-1 strains were classified in the C1C2 region of *env* as: CMNYU107: CRF36_cpx and subtype G; CMNYU6518: two CRF01_AE/CRF36_cpx URFs possessing inter-subsubtype (∼12%) genetic distance from one another; CMNYU129: CRF02_AG and subtype F2; CMNYU6544: CRF02_AG/F2 URF and CRF02_AG [37] (Table 1). The CD4 counts ranged from 134 to 603 cells/µl (mean 346 cells/µl) (Table 2). The time intervals of the plasma samples used for neutralization are presented in Table 2. From this same cohort, a total of 4 individuals identified as singly infected (with one subtype) were also studied and included CMNYU133 (subtype F2), CMNYU179 (CRF02_AG), CMNYU153 (subtype H), and CMNYU6542 (CRF02_AG) [38] (Table 1). The CD4 counts ranged from 204 to 923 cells/µl (mean 506 cells/µl) (Table 2). There were no significant differences in study duration or change in CD4 T cell count (ΔCD4) over the study period between the superinfected group and the singly infected control group (Table 2).

### RNA Extraction and RT-PCR Amplification of *gp120*

Viral RNA from patient plasma was extracted using the QIAamp Viral RNA Mini kit (Qiagen Inc, Valencia, CA) according to the manufacturer’s instructions. Two microliters of eluted RNA was used for reverse transcriptase polymerase chain reaction (RT-PCR) using the Superscript One-Step RT-PCR for Long Templates kit (Invitrogen, Carlsbad, CA), using primers EnvA (5′-GGCTTAGGCATCTCCTATGGCAGGAAGAA-3′) and gp120OUT (5′-GCARCCCCAAAKYCCTAGG-3′). The amplification conditions were as follows: one cycle at 50°C for 30 minutes and 94°C for 2.5 minutes, followed by 50 cycles at 94°C for 15 seconds, 50°C for 30 seconds and 68°C for 2.5 minutes, ending with a single extension cycle at 72°C for 7 minutes. Two microliters of first-round product was then used in a nested PCR using the Platinum PCR SuperMix High Fidelity system (Invitrogen, Carlsbad, CA), using the primers EnvB (5′-AGAAAGAGCAGAAGACAGTGGCA-3′) and gp120IN (5′-CGTCAGCGTYATTGACGCYGC-3′). For the second-round PCR, amplification conditions were: one cycle at 94°C for 2 minutes, followed by 35 cycles at 94°C for 15 seconds, 50°C for 30 seconds and 68°C for 1 minute. Two microliters of each PCR product was run on a 1% agarose gel to confirm amplification of the expected 1636bp fragment (HXB2 location 6202–7838) and the absence of any 1^st^ round products.

### Cloning, Sequencing and Phylogenetic Analysis

PCR products were cloned into the Topo TA cloning vector and plasmids were transformed into chemically competent *E.coli* cells according to manufacturer’s recommendations (Invitrogen, Carlsbad, CA). A total of 24 positive colonies, selected by Kanamycin resistance and X-Gal blue-white screening, were cultured in 3 ml LB broth plus 10 mg/ml Kanamycin overnight. Plasmids were purified using the QIAprep Spin Miniprep kit (Qiagen Inc, Valencia, CA). Positive clones were sequenced at the 5′ and 3′ end with the universal T3 and T7 primers. To obtain the full *gp120* sequence, primer walking was performed. Individual sequence fragments were assembled using the Pregap and Gap programs from the Staden software package [57] and open *gp120* reading frames were confirmed using the Sequence Locator tool provided by the Los Alamos HIV Sequence Database (http://www.hiv.lanl.gov/content/sequence/LOCATE/locate.html).

### Construction of Virus Pseudotyped with each Study Stubject’s HIV-1 *gp120*

Topo-cloned *gp120* sequences with open reading frames were used for creating *env*-expression vectors. Topo-cloned *gp120* and primers PseudoAmpF (5′-GAAGAGCAGAAGACAGTCGCGATGAAAGTGAAGGGGATAC-3′) and PseudoAmpR (5′-GTTATTGACGCCGCGCCCATAGTCGACCCTGCTGCTCCTAAGAAC-3′) were used to amplify *gp120* out of the vector while inserting NruI and SalI restriction sites, using the PCR SuperMix High Fidelity system, according to manufacturer’s instructions (Invitrogen, Carlsbad, CA). The amplification conditions were: one cycle at 94°C for 2 minutes, followed by 45 cycles at 94°C for 15 seconds, 50°C for 30 seconds and 68°C for 1.5 minutes. Two microliters of PCR product was run on a 1% agarose gel to verify correct amplification. The remaining product was then purified using the Qiaquick PCR Purification Kit according to manufacturer’s instructions (Qiagen, Valencia, CA), and subjected to a 2 hr NruI/SalI digest, followed by an overnight DpnI digest, according to manufacturer’s protocol (New England Biosciences, Ipswich, MA). Digested amplicon was then purified as above, quantified, and ligated into the p1930 vector using the T4 DNA Ligase kit according to manufacturer’s protocol (Invitrogen, Carlsbad, CA). The p1930 vector is a variant of the p166 vector (pcDNA3.1 backbone; kindly donated by Dr. Abraham Pinter), which expresses a subtype C HIV-1 envelope under the control of a CMV promoter. p1930 was created using Quickchange Mutagenesis (Stratagene, La Jolla, CA), which inserted an NruI site and a SalI site on the 5′ and 3′ ends, respectively, of the p166 *gp120*. Before ligation of the *gp120* amplicon into p1930, p1930 was subjected to NruI/SalI digest followed by overnight treatment with Calf Intestinal Alkaline Phosphatase (CIAP) and purification as described above. Chemically competent XL-1 Blue cells were transformed with the ligation reaction according to manufacturer’s protocol (Invitrogen, Carlsbad, CA), and plated on Ampicillin-LB plates. Colonies were screened by colony PCR for correctly ligated *gp120* insert, and sequenced as described above with primers RP-1770 (5′-GGCTTGCCTTAGGCATCTCC-3′) and RP-1773 (5′-GCTGTTGATCCTTTAGGTATC-3′) with primer walking. Sequence analysis was performed and sequences were checked for open reading frames as described above. Positive vectors were transformed into Stbl2 cells according to manufacturer’s protocol (Invitrogen, Carlsbad, CA), plated, and then one colony was picked and used to inoculate a 50 mL of LB-Ampicillin broth for overnight culture. Vector was purified from this culture using the HiSpeed Plasmid Midi kit according to manufacturer’s instructions (Qiagen, Valencia, CA).

### Cells

TZM-bl cells were used in neutralization assays (see below at “Titration and Neutralization assay of pseudotyped viruses” and [58]–[59]). These cells are maintained in DMEM supplemented with 10% fetal bovine serum, 1% glutamine, and 2% penicillin plus streptomycin (TZM-DMEM). The cultures were kept at 37°C in a 5%-CO_2_ humidified incubator.

### Pseudovirus Production

Purified vector was used to co-transfect 293T cells with the Q23Δ*env* HIV-1 backbone plasmid using Fugene 6, according to manufacturer’s protocol (Roche, Manheim, Germany) [60]. After 48 hours, supernatant was removed from cells, and spun at 3000 rpm for 10 min. The FBS concentration was brought up to 20% and the pseudovirus was stored at −80°C until use.

### Titration and Neutralization Assay of Pseudotyped Viruses

Pseudovirus was titrated in a 96-well plate, using 200 µl of viral supernatant in the first well and setting up a 2-fold dilution series with DMEM media along the row. Per well, 10 000 TZMbl cells were added in a volume of 100 µl, and DEAE-dextran was added to each well at a final concentration of 12.5 µg/ml. The plates were incubated for 72 h at 37°C. Then luminescence was measured using the Bright Glo Reagent (Bright-Glo Luciferase Assay System, Promega, Madison, Wisconsin) on a Lumimark Plus System microplate reader (Bio-Rad Laboratories, Hercules, CA) as previously described [38]. The titrated pseudoviruses were then tested in neutralization assays with TZM-bl cells, as previously described [58]–[59]. Briefly, 1∶20–1∶2560 diluted heat-inactivated plasma from each patient at each time point and a fixed dilution of each patient’s corresponding autologous pseudovirus were mixed in equal volumes (50 µl each) and incubated for 1 hour at 37°C; 10 000 cells in 100 µl of DMEM media containing 25 µg/ml DEAE-dextran (final concentration 12.5 µg/ml) was added to each well, and cells were incubated for 48 hours as previously described [38]. To measure the background luminescence, wells with cells only (no pseudovirus, no plasma) were included. As control, virus-only wells (cells infected with pseudovirus, no plasma) were also tested. Cells were lysed using the Bright Glo Reagent (Bright-Glo Luciferase Assay System, Promega, Madison, Wisconsin) and relative light units (RLU) were measured on a Lumimark Plus System microplate reader (Bio-Rad Laboratories, Hercules, CA) as previously described [38].

### Statistical Analysis, Calculation of Percent Neutralization, IC_50_ Titer, and Breadth-Potency Scores

Neutralization was assessed in duplicates, and experiments were repeated a minimum of two times. Percent neutralization was determined by dividing the mean RLU for each set of duplicates by the mean RLU in the appropriate replicates of virus-only control wells, after subtraction of the cells-only background from all RLU values. Mean IC_50_ values were determined by identifying the adjacent dilutions being above and below the 50% readout. The assay readouts for the dilutions above and below 50% neutralization were joined with a straight line and the position where the line crossed the 50% readout was taken as IC_50_ titer [61]. Statistical significance of overall pairwise neutralization comparisons was determined using the Mantel-Cox logrank test. The Wilcoxon rank sum test was used to compare the CD4 counts and viral load data between singly and superinfected patient groups. Breadth-Potency (B-P) scores, a measure that accounts for both the simultaneous potency and the breadth of a tested plasma sample, were determined as follows: A Breadth-Potency (B-P) curve was plotted where y  =  the fraction of viruses whose IC_50_ exceeds x, where x = log10 (IC_50_). At x = 0 the height is y =  fraction of viruses in the panel for which an IC_50_ was achieved, i.e. the “fraction neutralized”. The B-P score represents the area under the B-P curve, an area that increases with both breadth and potency. It can be shown that this area – the B-P Score – can be computed by simply averaging all x values obtained by setting x = log10(IC_50_) for the available IC_50_s and by setting x = 0 for viruses that did not neutralize. This is equivalent to multiplying the fraction of viruses neutralized by the mean of the available IC_50_s [40].
